# Lignin-Containing Cellulose Nanofibrils from TEMPO-Mediated Oxidation of Date Palm Waste: Preparation, Characterization, and Reinforcing Potential

**DOI:** 10.3390/nano13010126

**Published:** 2022-12-26

**Authors:** Amira Najahi, Quim Tarrés, Pere Mutjé, Marc Delgado-Aguilar, Jean-Luc Putaux, Sami Boufi

**Affiliations:** 1 LMSE, Faculty of Science, University of Sfax, Sfax BP 802–3018, Tunisia; 2 LEPAMAP-PRODIS Research Group, University of Girona, C/Maria Aurèlia Capmany, 61–17003 Girona, Spain; 3 Université Grenoble Alpes, CNRS, CERMAV, 38000 Grenoble, France

**Keywords:** lignocellulosic nanofibers, nanocellulose, TEMPO-mediated oxidation, property

## Abstract

Lignin-containing cellulose nanofibrils (LCNFs) have emerged as a new class of nanocelluloses where the presence of residual lignin is expected to impart additional attributes such as hydrophobicity or UV-absorption. In the present work, LCNFs with a lignin content between 7 and 15 wt% were prepared via a TEMPO-mediated oxidation as chemical pretreatment followed by high-pressure homogenization. The impact of the carboxyl content (CC) on the properties of the resulting LCNF gel, in terms of lignin content, colloidal properties, morphology, crystallinity, and thermal stability, were investigated. It was found that lignin content was significantly decreased at increasing CC. In addition, CC had a positive effect on colloidal stability and water contact angle, as well as resulting in smaller fibrils. This lower size, together with the lower lignin content, resulted in a slightly lower thermal stability. The reinforcing potential of the LCNFs when incorporated into a ductile polymer matrix was also explored by preparing nanocomposite films with different LCNF contents that were mechanically tested under linear and non-linear regimes by dynamic mechanical analysis (DMA) and tensile tests. For comparison purposes, the reinforcing effect of the LCNFs with lignin-free CNFs was also reported based on literature data. It was found that lignin hinders the network-forming capacity of LCNFs, as literature data shows a higher reinforcing potential of lignin-free CNFs. Nonetheless, the tensile strength of the acrylic matrix was enhanced by 10-fold at 10 wt% of LCNF content.

## 1. Introduction

Date palm is a flowering plant from the palm family, and it is widely cultivated in the Middle East and North Africa (MENA). The date palm tree can grow in desert environments, characterized by scarcity of rainfall, water shortage, and extreme temperatures. In addition, date palm is known to tolerate salinity more than other cultivated fruit crops, allowing its growth in coastal areas [1]. While date palm can be cultivated in all continents of the world, the MENA region accounts for 89% of date production. Concretely, Tunisia is one of the leading date palm producers and exporters, with an average yearly production of about 120 thousand tons derived from more than four million date palm trees. According to the Food and Agricultural Organization (FAO), Tunisia represented 10% of the global production in 2018 [2].

The date palm biomass contains a relatively high cellulose content in comparison to other lignocellulosic resources, which confers to the fibers derived from this resource good mechanical properties [3]. The agricultural activity related to date exploitation generates a large amount of waste in the form of date palm rachis and leaves that are usually left in the field or even burned. The cellulose content of leaves and rachis are around 55 and 40 wt%, respectively, and their hemicellulose contents account for approximately 20 and 28 wt% [4]. Further, the lignin content of date palm biomass usually ranges between 22 and 32 wt% [5]. This chemical composition offers to date palm wastes (DPWs) a myriad of opportunities in different sectors, such as biocomposites, but also other groundbreaking areas demanding biosourced and environmentally friendly materials and products [3,6,7,8]. In fact, one of the most promising applications for DPWs is the production of nanocellulose, particularly in the form of lignin-containing cellulose nanofibrils (LCNFs). Indeed, the production of LCNFs from DPWs is doubly advantageous. On the one hand, wastes can by highly valorized and negative impacts to the environment are potentially prevented, apart from promoting and adding value to agricultural activities. On the other hand, the excellent characteristics of DPWs and the preservation of lignin during the production of LCNFs can represent a strong alternative to wood-derived nanocellulose, as similar performance can be achieved, while valorizing an agricultural waste and, thus, contributing to alleviating the pressure over forests [9].

The production of LCNFs from various sources by means of different production pathways is a topic of great interest due to the low generation of residues during the process, as well as the unique characteristics of the LCNFs [9,10,11,12]. One of the main advantages of LCNFs against cellulose nanofibrils (CNFs) is the higher operation yields of the former since lignin is preserved along the process [11]. In addition, the presence of lignin may be beneficial in terms of surface charge, polarity, and chemical functionalization, apart from omitting the bleaching steps, which may result in lower production costs [13,14]. In addition, the presence of lignin and hemicelluloses has been reported to promote fibrillation, which may result in a decrease in the energy consumption during the fibrillation stage [12]. Indeed, the literature reports several strategies to decrease this energy consumption by means of the so-called pretreatments. These pretreatments usually consist of either chemical, enzymatic, or mechanical treatments that avoid clogging in the fibrillation equipment [15]. The literature is extensive on these pretreatments, although the mechanisms remain under study, mainly due to the high heterogeneity of the raw materials and the lack of a common understanding of the effect of fiber recalcitrance [16,17].

In terms of pretreatments for LCNF production, mechanical refining is the most widely used [14,18]. After appropriate fiber isolation by means of traditional processes (i.e., organosolv, kraft, sufite, or NaOH-anthraquinone), the resulting fibers are usually highly refined to promote their swelling and length reduction. This process is energy-intensive and the resulting LCNFs exhibit lower characteristics than other grades of CNFs, particularly those prepared by means of chemical or enzymatic processes [19]. Another strategy consists in incorporating specific enzyme cocktails, mainly composed by glucanases, that impart a regioselective treatment over the cellulose chain, mostly onto the β-1,4 glucosidic bonds, but also in other regions due to the heterogeneity of commercial enzyme cocktails [20,21]. However, the presence of lignin may limit the accessibility of these enzymes to the cellulose chain due to steric effects, lowering the yield of the whole operation or, otherwise, requiring longer treatment times [22,23]. This lower accessibility of the enzymes in lignin-containing fibers was already observed by Espinosa et al. and significantly lower yields of fibrillation were found for enzymatic treatments compared to mechanical treatments [24]. Both pretreatments have been reported to have a minor effect on the chemical composition of the neat fibers, which clearly indicates that most of the initial resource is preserved. Nonetheless, while it is true that the presence of lignin and other amorphous constituents promote fibrillation during high-pressure homogenization, for instance, it is also worth noting that other nanostructured particles may be released, like lignin nanoparticles [25,26,27].

Regarding chemical pretreatments, TEMPO-mediated oxidation has appeared as one of the most promising pathways to produce CNFs, mainly starting from bleached fibers [28,29]. In the presence of lignin, the TEMPO/NaBr/NaClO oxidizing system acts on the carbon 6 (C6) of the cellulose chain (oxidation of the primary alcohol) while degrading lignin, resulting in two parallel reactive systems and, thus, decreasing the cellulose oxidation rate. As observed by Jiang et al., the presence of lignin and hemicelluloses hinders cellulose oxidation, as most of these amorphous constituents are degraded during the process [10]. In addition, the presence of residual lignin was reported to have negative effects on the nanodispersion. The influence of lignin on the TEMPO/NaBr/NaClO oxidation has previously been reported by other authors, and low carboxyl contents were found compared to bleached fibers [24]. In addition, the presence of lignin, due to its hydrophobic character, is assumed to impart negative effects over the gel formation.

Although TEMPO-mediated oxidation is not a good option for the production of LCNFs, the gradual oxidation of lignin-containing fibers by means of TEMPO-mediated oxidation could be beneficial to tailor the resulting characteristics of LCNFs. In this sense, although higher oxidizer dosages would be required for achieving a given content of carboxyl groups, the presence of lignin could be beneficial for specific applications [11,30,31]. In addition, the use of agricultural residues is of particular interest, as the generation of wastes from wastes would be minimized [32,33]. This has previously been reported for cereal crops and other residues such as sawdust with great opportunities in a myriad of applications. Further, although the literature on the use of DPWs for LCNF production provides a solid state of the art, the use of TEMPO-mediated oxidation of this residue without delignification has never been explored. Therefore, in this work we present an approach to prepare TEMPO-oxidized LCNFs from high-lignin content DPW fibers obtained by chemical-free methods and discuss the effect of carboxyl content on the gel forming capacity to obtain LCNF suspensions with similar gel characteristics to those obtained with CNFs prepared from bleached wood fibers.

## 2. Materials and Methods

### 2.1. Materials

Date palm wastes (DPWs) were obtained from the annual pruning of date palm trees in the region of Gabès (Gabès, Tunisia). 2,2,6,6-tetramethylpiperidine-1-oxyl radical (TEMPO), sodium bromide (NaBr), sodium hypochlorite solution (NaClO) were commercial products supplied from Sigma–Aldrich (Tunis, Tunisia). The NaClO concentration was checked by volumetric titration with sodium thiosulfate.

### 2.2. Preparation of LCNFs

LCNFs from DPWs were prepared using a three-stage process, encompassing biomass preparation, TEMPO-mediated oxidation, and high-pressure homogenization.

#### 2.2.1. Biomass Preparation

DPWs were first subjected to mechanical grinding using a blade mill to reduce the particle size to around 5–10 mm and were then passed through a Sprout-Waldron defibrator 105-A (Muncy, PA, USA) to defibrillate the biomass until obtaining coarse fibers.

#### 2.2.2. TEMPO-Mediated Oxidation of the DPWs

After mechanical defibration, DPWs (5 g) were suspended in 400 mL water. TEMPO (80 mg) and NaBr (500 mg) were added to the suspension. Then, the appropriate volume of a commercial NaClO solution (12 vol%) was added dropwise for 2 h to the DPW suspension, kept under mechanical stirring, and immersed in a glass bath to maintain the temperature around 4–8 °C during oxidation. The pH was maintained around 10 by the dropwise addition of a 0.1 M NaOH solution. After an additional stirring for 1 h to complete oxidation, the fibers were recovered by filtration and washed with demineralized water until the conductivity of the recovered water became close to its initial value. After the oxidation, the recovered material was yellowish to white with a fibrous aspect. Three different volumes of NaClO solution were used, namely 110, 170, and 220 mL, corresponding to 500, 832, and 1250 mmol g^−1^, respectively.

#### 2.2.3. High-Pressure Homogenization (HPH)

The suspension of oxidized fibers at a solid content around 1.5 wt% was pumped through a GEA Niro Soavi High-Pressure Homogenizer processor (NS1001L PANDA 2 K-GEA, Parma, Italy) by starting with a pressure around 300 bar (4350 Psi) during three passes until the suspension turned to a thin gel, and then the HPH was pursued at a pressure of 600 bar (8700 Psi) for four to six passes, to further disintegrate the fibers.

### 2.3. Carboxyl Content (CC)

The carboxyl content (CC) of the oxidized cellulose was determined by conductometric titration according to the method described elsewhere [34]. Briefly, about 100 mg of fibers were dispersed in 50 mL water, which was later acidified by means of the incorporation of few drops of a 0.01 M HCl solution, reaching a pH 3. Then, the suspension was titrated with a NaOH solution (0.005 M), using conductimetry to monitor the titration. Three samples of LCNF with a carboxyl content of 500, 832 and 1250 µmol/g were prepared. When referring to the LCNFs, they were labelled as LCNF-500, LCNF-800 and LCNF-1200, respectively, while when referring to the oxidized pulps, the term DPW was used (DPW-500, DPW-800, and DPW-1200). Results are presented as the average of three measurements.

### 2.4. Chemical Composition

The chemical composition was determined according to TAPPI standard protocols. Samples were first submitted to Soxhlet extraction with ethanol/toluene and water to evaluate the extractive content (Tappi T264 om-07). Ash and Klason lignin were determined according to TAPPI standards T211 om-93 and T222 om-88, respectively, while holocellulose was determined according to a previously reported method [35]. Tests were performed in duplicate.

### 2.5. Transmission Electron Microscopy (TEM)

Droplets of highly diluted LCNF suspensions were deposited on glow-discharged carbon-coated copper grids. Before drying, the preparations were negatively stained with 2% uranyl acetate. After 1 min, the stain in excess was blotted with filter paper and the remaining liquid film allowed to dry. The suspensions were observed with a JEOL JEM-2100 Plus transmission electron microscope operating at 200 kV. A Gatan Rio 16 camera was used to record the images.

### 2.6. Atomic Force Microscopy (AFM)

Specimens were prepared by depositing a drop of a LCNF suspension with a solid content about 0.01 wt% on the surface of a silicon wafer and leaving it to dry for 1 h. Images were recorded with a Nanosurf Flex atomic force microscope operating in tapping mode.

### 2.7. Fourier-Transform Infrared (FTIR) Spectroscopy

The FTIR-ATR spectra were collected with a PerkinElmer Spectrum II equipped with a diamond crystal plate ATR MIRacle Single Reflection Accessory, at a resolution of 4 cm^−1^ from 500–4000 cm^−1^.

### 2.8. Thermogravimetric Analysis (TGA)

The analysis was performed with a TGA 400 thermogravimetric analyzer from Perkin Elmer. Samples were about 10 mg, and the heating cycle was from 40 to 800 °C at a heating rate of 10 °C min^−1^ under air flow. Tests were performed in duplicate.

### 2.9. Particle Size and ζ-Potential Measurements

The particle size and surface charge were measured at 25 °C by dynamic light scattering (DLS) using a Zetasizer ZS apparatus from Malvern. The concentration of the LCNF suspension was kept around 0.2% and pH controlled by NaOH/HCl (10^−3^ M). Each measurement was performed in triplicate.

### 2.10. Rheological Measurements

The rheological measurements were carried out at 25 °C and pH around 7, using a stress-controlled Rheometer (Kinexus Pro+, Malvern Instruments, Malvern, UK) with a cone plate geometry (cone angle, 2°; diameter, 20 mm; truncation, 56 µm). Dynamic mode tests were performed by first measuring the storage modulus G’ and the loss modulus G” vs. strain to determine the linear behavior domain. Then, a frequency sweep at a fixed strain in the linear domain was performed. Each measurement was performed in triplicate.

### 2.11. Nanocomposite Processing

A commercial acrylic latex (S330 from MPC-Prokim-Tunisia), obtained by the copolymerization of styrene (Sty) and butyl acrylate (BuA), was used as a matrix. The size of the polymer particles was around 200 nm and the solid content, 50 wt%. The glass–rubber transition temperature (Tg) of this poly(Sty-co-BuA) copolymer was around 15 °C, according to the supplier. The nanocomposite films were prepared by solvent casting. Briefly, the appropriate amount of LCNF gel was mixed with the latex at a weight fraction ranging from 0 to 10 wt%. After stirring for 1 h, the mixture was cast in a Teflon mold and stored at 40 °C until water evaporation and film formation. A transparent to translucent film, depending on the LNFC content, was obtained with a thickness in the range of 200 to 300 µm.

### 2.12. Tensile Tests

The tensile-test measurements were run using an Instron testing machine in tensile mode, with a load cell of 100 N working at a strain rate of 10 mm min^−1^ at 25 °C. The specimens were obtained using a cutting device. Results are presented as the average of five tests.

### 2.13. Transmittance of the Films

The transmittance of both the neat acrylic film and the nanocomposite films was measured using a UV-visible spectrophotometer (Lambda 35, Perkin-Elmer, Waltham, MA, USA) operating in the wavelength range of 400 to 800 nm. The transmission spectra of the films were recorded using air as reference. Each measurement was performed in triplicate.

### 2.14. Dynamic Mechanical Analysis (DMA)

DMA experiments were conducted using a PYRISTM Diamond DMA (Perkin-Elmer, Waltham, MA, USA). Temperature scans were run from 30 to 100 °C at a heating rate of 2 °C min^−1^, a frequency of 1 Hz and an amplitude of 10 μm. Results are presented as the average of five tests.

### 2.15. Contact Angle Measurement

Contact angle measurements were carried out by depositing calibrated liquid drops on the surface of thin films using an OCA 15 Drop Shape Analyzer from Dataphysics, equipped with a high-resolution CCD camera, working at an acquisition of 50 images per second. Thin films were prepared by casting 0.5 wt% suspensions in a Petri dish and drying at 40 °C until complete evaporation of water. Results are presented as the average of five tests.

### 2.16. X-ray Diffraction (XRD)

The XRD profiles of the DPW and LCNF films were recorded in a Warhus vacuum chamber using a Philips PW3830 generator operating at 30 kV and 20 mA (Ni-filtered CuKα radiation, λ = 0.1542 nm), during 1h exposures. The crystallinity index (CrI) was estimated using Segal’s equation (Equation (1)).

(1) $$CrI=(\frac{I_{Crs}-I_{am}}{I_{Crs}})$$

where I_Crs_ is the intensity of the 200 peak at 22.3° and I_am_, the intensity of the minimum between the 200 and 110 peaks at 18°. Tests were performed in duplicate.

## 3. Results and Discussion

### 3.1. Chemical Composition of the TEMPO-Mediated Oxidized DPWs

As detailed in the previous section, the oxidation of DPWs was conducted with three different amounts of oxidizer. The CC of the fibers reached about 500, 832, and 1250 µmol g^−1^, when the oxidation was run in presence of 6, 10, and 13 mmol NaClO/g DPW, respectively. A significant evolution of the aspect of the fibers as well as their chemical composition was noted after the oxidation treatment. The fibers with a CC of 1250 µmol g^−1^ were apparently more individualized and looked white, with an apparent high swelling capacity, which was presumably due to the bleaching effect of NaClO, while those with a CC of 500 and 832 µmol g^−1^ were yellowish, with less individualized fibers, mostly at a CC of 500 µmol g^−1^.

**Figure 1:**
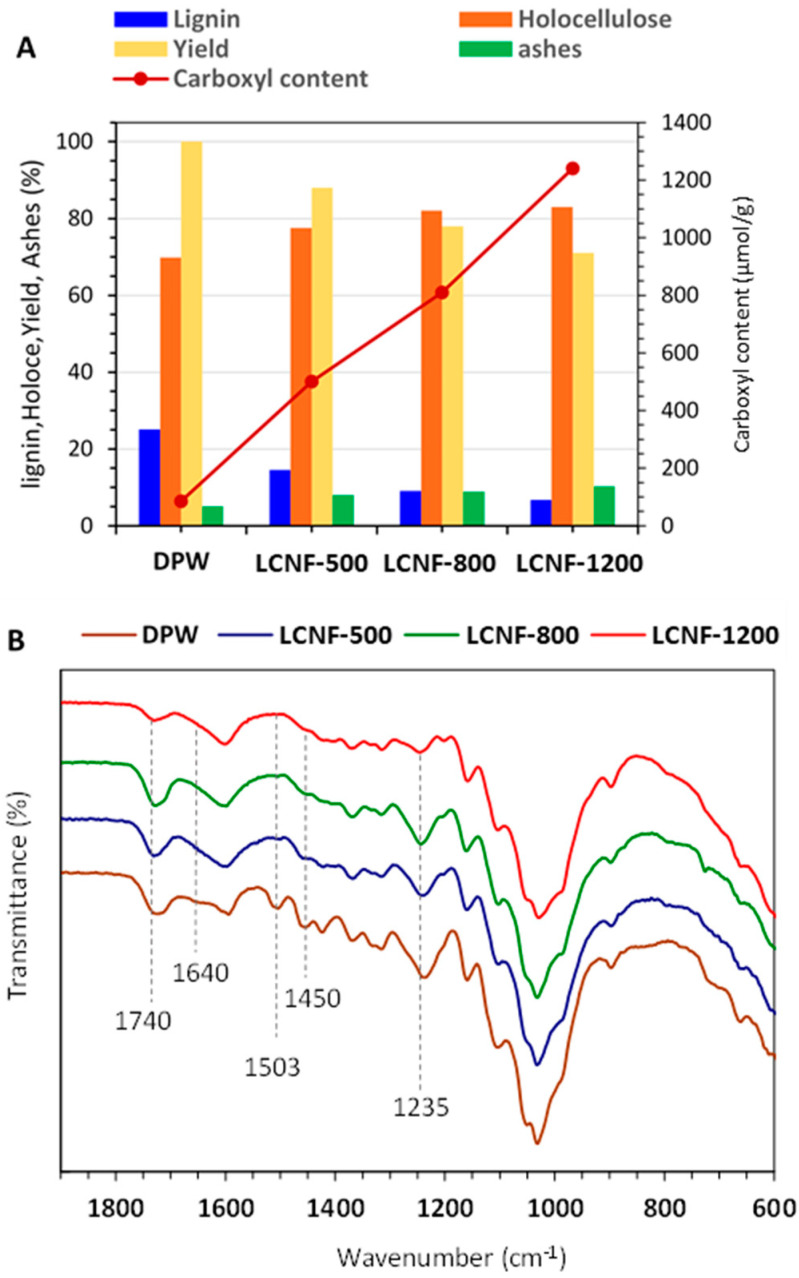
(**A**) Carboxyl, lignin, and holocellulose contents, and yield in recovered fibers for the different TEMPO-oxidized DPW. (**B**) FT-IR spectra of the original DPWs and the different TEMPO-oxidized samples.

As shown in Figure 1, the lignin content markedly decreased with increasing CC, dropping from about 26 wt% for the neat DPW to 14.5, 9.1, and 6.7 wt% for DPW-500, DPW-800, and DPW-1200, respectively. This decrease in lignin content was accompanied by a decrease in the yield of the treatment, accounting for 88, 78, and 71%, at increasing CC. This tendency was expected and in line with the literature, where it was demonstrated that the TEMPO-mediated oxidation of thermomechanical pulps is accompanied by a degradation and dissolution of lignin in the pulp [36]. In addition, it was postulated that the carboxylate groups are formed not only from the C6 primary hydroxyls of cellulose and hemicelluloses but also from lignin during the oxidation process [37]. Further confirmation of the reduction in lignin content was provided by FTIR analysis, where the major lignin absorption bands at wavenumbers of 1645, 1503, 1450, and 1235 cm^−1^, present in the original DPWs, which decreased in intensity in oxidized samples and even vanished at 1503 cm^−1^ for the highest CC; this further confirmed the removal of lignin as water-soluble compounds during the oxidation process. This result agrees with other works concerning the preparation of LCNFs from mechanical pulp via a TEMPO-mediated oxidation [38]. Considering that samples for FT-IR measurement were prepared from the oxidized pulp at pH 10, all the carboxyl groups were in the form of sodium carboxylate groups in the TEMPO-oxidized fibers, which could explain the decrease in the intensity of the C = O absorption band at 1740 cm^−1^.

### 3.2. LCNFs from TEMPO-Oxidized DPWs

**Figure 2:**
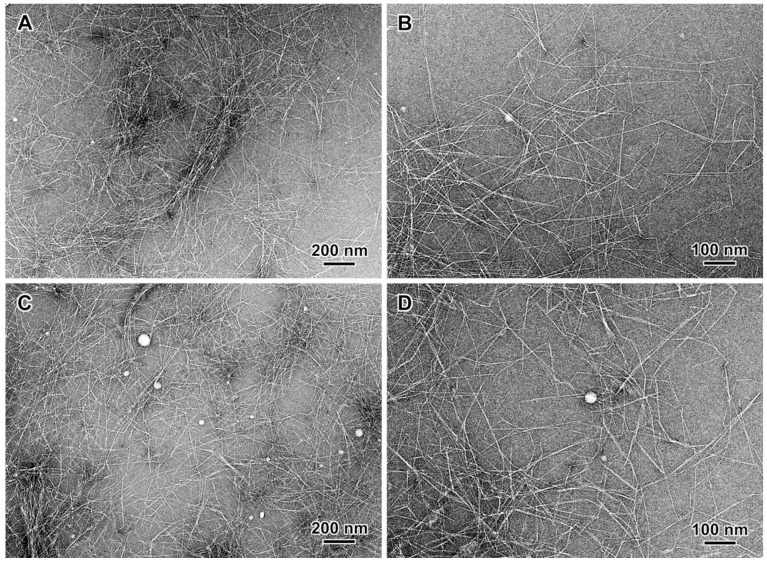
TEM images of negatively stained preparations from the supernatant fractions of LCNF-800 (**A**,**B**), and LCNF-1200 (**C**,**D**).

DPW samples were easily disintegrated into LCNF after six passes through the high-pressure homogenizer regardless of the oxidation degree. The three LCNF samples (LCNF-500, LCNF-800, and LCNF-1200) exhibited a thick gel-like aspect at 1 wt% consistency and were translucent for LCNF-500 and LCNF-800, and was quite transparent for LCNF-1200, suggesting an effective breakdown of the oxidized fibers into nanoscale for the highest CC. Their color is yellowish to light brown, depending on the lignin content: the higher the lignin content the more yellowish is the color, as it will be later discussed. The TEM images of negatively stained LCNF preparations (Figure 2 and Figure S1) revealed bundled and individualized slender ca. 3 nm wide nanofibrils forming entangled networks. White spherical particles could also be seen, which presumably correspond to lignin nanoparticles (NPs). The location of residual lignin in LCNFs is still a matter of debate. Depending on the preparation route of LCNFs, lignin can form patches attached to the LCNF fibrils [39], or in the form of spherical NPs detached from the fibrils [10].

**Figure 3:**
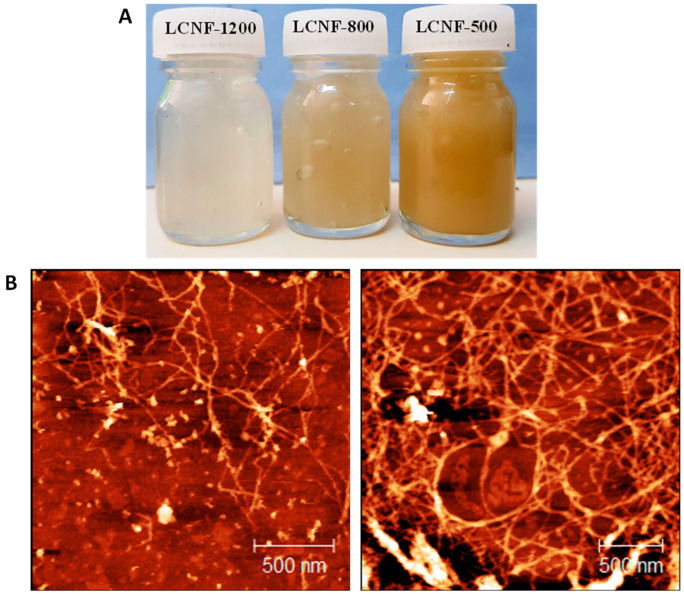
(**A**) Visual appearance of the LCNF suspensions. (**B**) AFM height images of LCNF-800.

Presently, we infer that the lignin is present in the form of free NPs in suspension with the cellulose nanofibrils, but also attached to the CNFs, as highlighted from AFM observations (Figure 3B). The difference in the morphology of LCNFs according to the observation mode TEM/AFM might arise from the specificity of each of them, as the lateral resolution of AFM is lower than TEM. While the analyzed TEM preparations correspond to the supernatant fraction of the diluted suspension, in AFM, the surface of a sample prepared by drying a drop of suspension on a flat wafer surface was observed.

**Figure 4:**
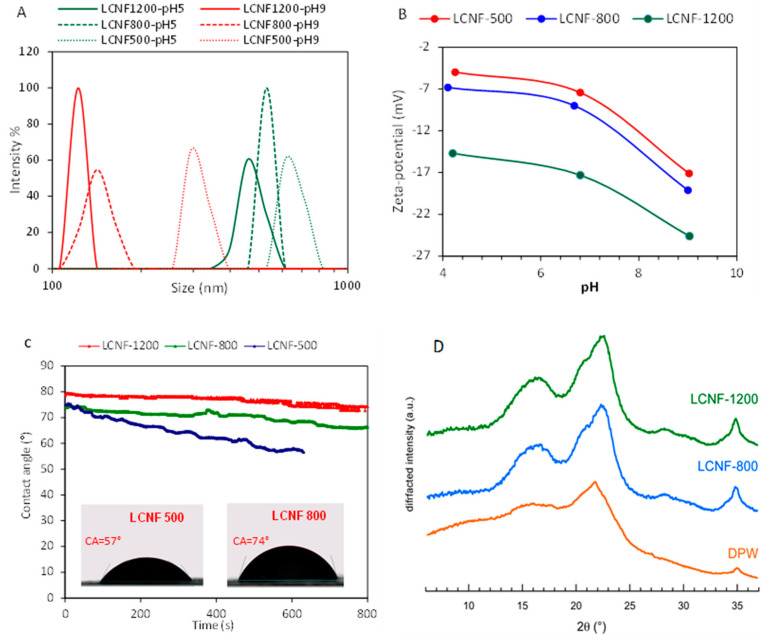
(**A**) Particle-size distribution of LCNFs at pH 5 and pH 9. (**B**) ζ-potential vs. pH of LCNFs. (**C**) Contact angle of water vs time on LCNF films. (**D**) X-ray diffraction profiles of DPW, LCNF-800, and LCNF-1200 thin films.

The colloidal properties of the LCNFs were investigated by DLS and ζ-potential measurement at different pH and rheological analysis. DLS size calculation is based on the measurement of the hydrodynamic diameter of a sphere with an equivalent translational diffusion coefficient as the particle. Since our objects in suspension are fibrillar, the calculated value is thus not meaningful. However, the technique was used to compare the systems in terms of size distribution and colloidal stability as a function of pH and CC. The size distribution was the lowest for LCNFs with the highest CC (LCNF-1200) and was shifted to a higher size with decreasing pH from 9 to 5. This confirms that the colloidal stability of LCNFs is increased at basic pH, mainly due to the ionization of the carboxyl groups providing negative charges on the LCNF surfaces that oppose aggregation through electrostatic repulsion. This was confirmed from ζ-potential measurement since the absolute value of ζ-potential at a given pH increased with CC and was the highest at pH 9 (Figure 4B).

The surface properties in terms of hydrophilic/hydrophobic characteristics were assessed by contact angle measurement performed on a thin dried film of the LCNF, obtained by casting and drying at room temperature. The evolution of the contact angle with time of a calibrated water droplet is shown in Figure 4C. The highest contact angle was observed for LCNF-1200, with an initial contact angle θ_0_ = 80° followed by LCNF-800 and LCNF-500 with θ_0_ around 75°. The contact angle remained nearly the same for LCNF-1200 and LCNF-800 films, while it clearly decreased with time for LCNF-500, presumably due to the absorption of the water droplet by the film. The relatively high contact angle for LCNF-1200 and LCNF-800 films indicates a hydrophobic surface, which is likely due to the presence of residual lignin known to impart a hydrophobic character to the LCNFs, as pointed out in the literature data [12,40]. It is worth mentioning that the contact angle for a film of fully delignified CNFs, and originating from the same DPW biomass, exhibited a contact angle of 45° (data not shown), which agrees with literature data concerning the contact angle for lignin-free nanopapers [41]. However, it is surprising to achieve the highest contact angle for the LCNF-1200 film, despite its lowest lignin content (around 6 wt%), in comparison with LCNF-800 or LCNF-500 containing higher lignin, around 9 and 14 wt%, respectively. It is also worth mentioning that the contact angle values for nanopapers or thin films from LCNFs, were systematically higher than those from lignin-free CNF films, but with a wide range in the values (extending from 60 to more than 95°) [42]. This disparity in contact angle values for LCNF nanopapers is likely due to multiple parameters controlling the contact angle for thin film, including, surface functionality, porosity, surface roughness, and chemical composition.

The XRD profiles of DPW and LCNF-800 and LCNF-1200 in the form of thin film are shown in Figure 4D. The LCNF samples yielded the typical diffraction peaks of cellulose I with a broad peak centered at 2θ = 16.2° corresponding to the merged 1$\bar{1}$0 and 110 reflections, and two peaks at 22.4 and 34.6° associated with the (200) and (004) crystal planes, respectively (indexing for the Iβ allomorph) [43]. This was expected as the TEMPO-mediated oxidation of cellulose under basic or neutral conditions does not alter the allomorphic type of cellulose fibers. However, although some of these features can be recognized in the profile of neat DPW, the main peak is located at 2θ = 21.8° and is not a reflection from cellulose. It likely results from the presence of epicuticular wax [44]. Indeed, this increase on crystallinity is attributed to the removal of amorphous fractions from the fibrils [45]. The crystallinity of the samples increased upon removal of the waxy constituent and a fraction of lignin during TEMPO-oxidation. LCNF-800 and LCNF-1200 exhibited nearly the same CrI of 0.61 as the lignin content in the two samples did not significantly differ.

**Figure 5:**
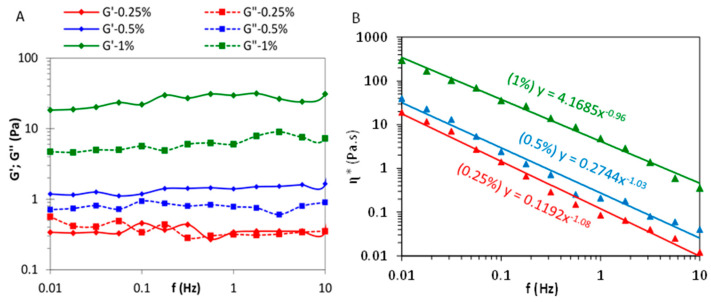
(**A**) Frequency sweeps of G’ and G” for LCNF-1200 at different solid contents (1, 0.5 and 0.25 wt%), and (**B**) the corresponding complex viscosity.

The rheological behavior of the LCNF gel was studied for LCNF-800 at different solid contents and under dynamic conditions by oscillatory sweep measurements of the storage modulus (G′) and loss modulus (G″) as a function of frequency (f) in the linear domain (Figure 5A). At a solid content (SC) of 1.0 and 0.5 wt%, the LCNF gel exhibited a gel-like character, as attested by the prevalence of G′ over G″, which is indicative of the of the generation of interconnected three-dimensional network among the nanofibrils. At 0.25 wt%, G′ decreased by about two orders of magnitude as a result from the weakening of the LCNF network due to the reduction in the number of fibril–fibril contacts upon dilution with water. The assessment of the complex viscosity (η*) at different frequencies (f) (Figure 5B) showed a shear thinning behavior at all SCs, as viscosity was found to decrease with increasing frequency. A power-law dependence of the η* vs f (η* = K.f^n^) was found, with n power index nearly close de -1, which is in line with the gel-like aspect of the LCNF suspension. This decrease in viscosity is expected for fibrils forming entangled networks and resulted from the progressive breakdown of the LCNF network under the effect of shear. In addition, the viscosity increased with SC, which further confirmed the network-like structure in the suspension.

**Figure 6:**
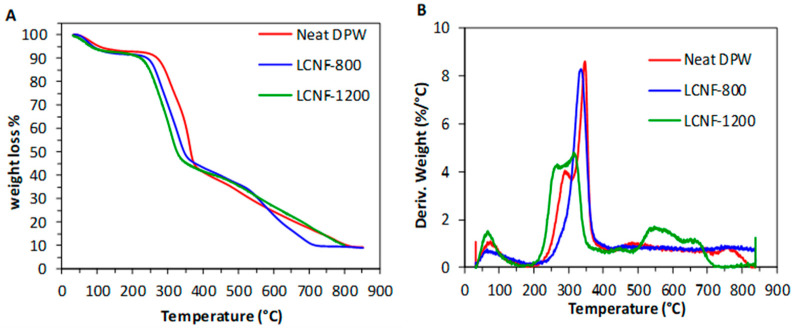
Thermogravimetric (**a**) and the corresponding derivative thermogravimetric (DTG) (**b**) curves of the original DPWs, LCNF-800, and LCNF-1200, under air atmosphere.

The thermal stability of neat DPWs and LCNFs were studied by TGA under air flow (Figure 6). Both thermograms displayed three weight losses. The first one, between 90–100 °C, with a weight loss around 8%, is attributed to water removal from ambient humidity. The second one, starting around 230 to 260 °C, depending on the composition, up to 350 °C, with a weight loss of about 50%, corresponds to the thermal degradation of hemicellulose and cellulose. This process involved the cleavage of the glycosidic bonds, the depolymerization and the dehydration of glycosidic rings. The thermal degradation started at 230, 245, and 265 °C, for LCNF-800, LCNF-1200, and DPWs, respectively, meaning that the higher the carboxyl content in the LCNF, the lower the onset of thermal degradation. This behavior was also observed for TEMPO-oxidized CNFs, where the presence of sodium carboxylate groups decreased the onset temperature to 220 °C from approximately 275 °C for the original cellulose, which is due to the decarbonation of the anhydroglucuronate [46]. The presence of lignin in the LCNFs may explain the higher thermal stability for LCNFs with lower CCs, and thus higher lignin content.

### 3.3. Mechanical and Thermal Properties of Nanocomposite Films

**Figure 7:**
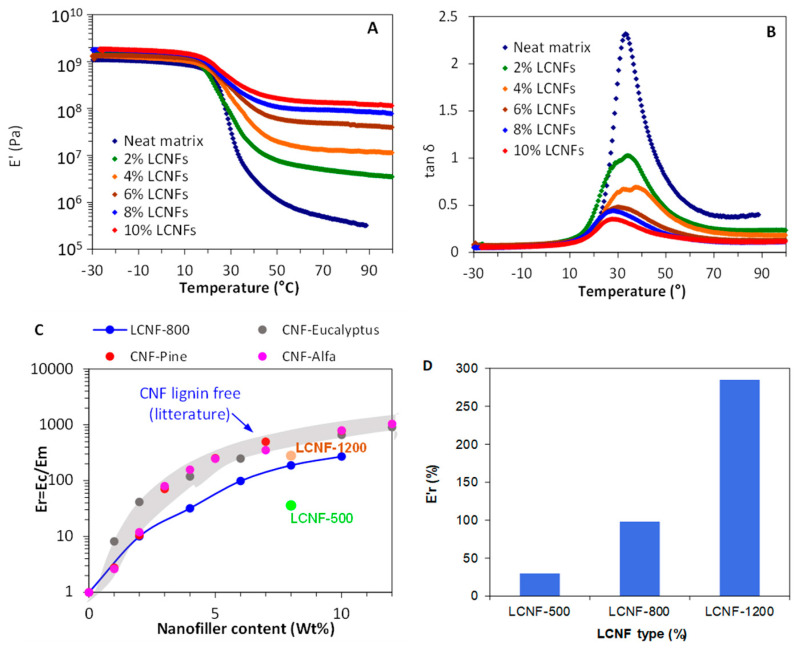
(**A**) Variation of storage modulus *E’* and (**B**) tan δ with temperature for nanocomposite films at different LCNF-800 contents. (**C**) Plot of *E_r_* vs. LCNF-800 content for the nanocomposite films at 70 °C compared to data from the literature on acrylic containing lignin-free CNFs. (**D**) *E_r_* at 70 °C for nanocomposite films containing 6 wt% LCNF-500, LCNF-800, and LCNF-1200.

**Figure 8:**
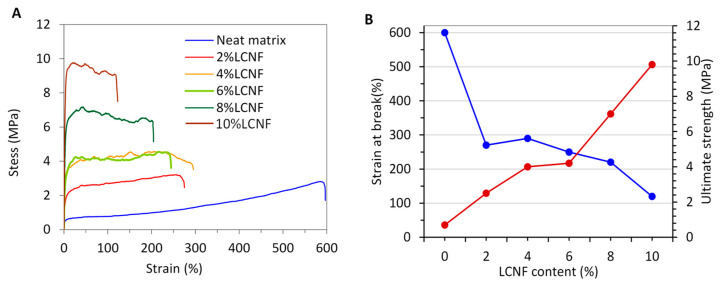
(**A**) Stress-strain plots of neat matrix and nanocomposites films at different LCNF-800 contents and (**B**) evolution of both strain at break and ultimate strength as function of the LCNF content.

One of the potential uses of LCNFs is as reinforcing nanofiller for ductile polymer matrices, mostly when the polymer is in the form of a waterborne latex. This allows the processing of the nanocomposite by mixing the nanocellulose suspension in water with the latex, followed by casting or coating of the mixture. The film-forming ability of nanocellulose-latex suspensions promotes the generation of a thin layer after water removal or absorption by the substrate. To investigate the reinforcing potential of the LCNFs, nanocomposite films containing between 2 and 10 wt% LCNFs were prepared following the methodology described in the experimental section and using an acrylic polymer latex dispersion. The mechanical properties of the resulting nanocomposite films were studied by DMA and stress-strain analysis (Figure 7 and Figure 8).

The temperature dependency of the storage modulus (*E′*) of the nanocomposite films with different LCNF contents is shown in Figure 7A. The neat matrix exhibited the typical behavior of an amorphous polymer with a *T_g_* around 30 °C, which usually shows a three-order of magnitude decrease of *E′* between 20 to 40 °C, and a maximum in the tan δ trace around 30 °C (Figure 7B). In the case of the films containing LCNFs, an upward shift of the *E′* trace was observed, indicating an increase in the film stiffness in the glassy and rubbery domains, with an effect much more pronounced in the rubbery domain. This behavior is typically observed in CNC- and CNF-filled polymer matrices and it is explained by the aptitude of the nanocellulose fibrils to set up a percolated and entangled network in the whole nanocomposite volume, mainly induced by hydrogen bonding, over a critical threshold [47]. To further highlight the reinforcing effect of the LCNFs, the increment in the moduli (*E_r_′*) was determined as the ratio between the storage moduli of the nanocomposites (*E’_c_*) and the matrix (*E’_m_*), both measured in the rubbery region at 70 °C. Figure 7C clearly reveals that the inclusion of LCNFs in the acrylic matrix strongly enhanced the stiffness of the film, as attested by the steady increase in the *E_r_′* with LCNF content. For instance, at 4 and 10 wt% LCNF content, the moduli of the nanocomposite films were, respectively, about 30 and 270 times higher than that of the neat matrix. This indicates that at 9 wt% lignin content, the reinforcing potential of the LCNFs is still preserved. The aptitude of LCNFs to convey a reinforcing effect was also for other types of polymer matrices, namely PVA [48] and PLA [3]. However, when comparing the reinforcing effect at 8 wt% nanofiller for the LCNFs with different lignin content (Figure 7C), it was noted that the stiffening effect was highest for LCNF-1200, followed by LCNF-800 and LCNF-500. Even though further research is needed to confirm this tendency, it appears that the reinforcing potential of the LCNFs is strongly correlated with their lignin content, decreasing at increasing lignin contents in LCNFs. One possible reason for this effect is the reduction in the cohesion of the fibril networks by the presence of residual lignin, either in the form of NPs or patches attached to the LCNFs. This would reduce the aptitude of the LCNFs to set up strong interactions through hydrogen bonding between entangled fibrils. Another important aspect is the lower reinforcing effect of the LCNFs in comparison with lignin-free CNFs. Indeed, regarding the evolution of *E_r_′* in presence of CNFs from different origins (data collected from previous works) [49,50], although all of them were produced from lignin-free cellulose fibers and using the same acrylic matrix, it can be seen that the domain of *E_r_′* for the CNFs was above that of the LCNF trace, mostly over a nanofiller content of 2% (Figure 7C). This further supports the hypothesis that the presence of lignin in LCNFs reduces the reinforcing potential when included in a polymer matrix. Nonetheless, further research is required in this direction, as some works highlighted that moderate amounts of lignin may contribute to their distribution within the polymer matrix [51].

A tensile-strain analysis of the nanocomposite films was also conducted as a complement to assess the reinforcing potential of the LCNFs. This mechanical analysis was run under non-linear regime and provided, in addition to the tensile modulus (E), the tensile strength (*σ*), and the elongation at break (ε). The stress–strain profiles of the nanocomposite films at different LCNF contents are presented in Figure 8A, and the corresponding ultimate strength (*σ_u_*) and strain at break (ε) are presented in Figure 8B. Comparatively to the neat acrylic film, the tensile strength of the nanocomposites was enhanced by the inclusion of LCNFs, while the strain at break notably decreased. This means that the acrylic film containing LCNFs was tougher and more resistant, with an effect that is proportional to the LCNF content. This confirms that the presence of lignin is limiting the percolation and network-forming capacity of LCNFs, as demonstrated in previous works; we observed that the presence of an entangled network may contribute to the ductility and toughness of the materials, or, at least, the negative effect becomes lower [52,53]. Lignin, in moderate amounts, has been reported to promote the stress transfer between individual fibrils, mainly due to its function as binder or crosslinking agent among them [54,55].

## 4. Conclusions

In the obtained LCNF suspensions in this work, lignin contents exhibited a thick gel-like aspect at 1 wt% solid content, with a translucid-yellowish aspect due to the presence of lignin. The lignin content decreased at increasing oxidation degrees and was attributed to the degradation and dissolution of lignin during the TEMPO-mediated oxidation. This evolution was confirmed by chemical and FTIR analyses. TEM images of LCNFs revealed more or less individualized nanofibrils forming an entangled network. The colloidal stability of the LCNFs was the highest over pH 7, where most of the carboxyl groups were ionized, providing negative charges that opposed aggregation through electrostatic repulsion. The presence of lignin in the LCNFs conveyed a hydrophobic character to the LCNFs, as confirmed by the high contact angle of thin film from dried LCNFs exceeding 70°. From TGA analysis, the onset of thermal degradation started at a temperature between 230 to 265 °C, depending on the CC. The higher the lignin content, the higher the thermal stability. The reinforcing potential of the LCNFs incorporated into a polymer latex matrix was also investigated by DMA and tensile-test analysis run on nanocomposite films by casting. The inclusion of LCNFs in the acrylic matrix was found to strongly enhance the stiffness and strength of the film, leading to a 10-fold increase in the case of tensile strength. However, when compared to lignin-free CNFs, a lower reinforcing effect was noted, indicating that the presence of lignin in the LCNFs reduced their reinforcing potential. This lower reinforcing potential was attributed to the lower network-forming capacity of LCNFs compared to CNFs, mainly due to the presence of lignin, which limits the interactions between individual fibrils.

## Data Availability

Data can be made available upon request.

## Supplementary Materials

The following are available online at https://www.mdpi.com/article/10.3390/nano13010126/s1, Figure S1: High-magnification TEM images of negatively stained preparations from LCNF-800 (A) and LCNF-1200 (B) showing bundled and individual cellulose nanofibrils.
