# Supplementary material for: Lignin-Containing Cellulose Nanofibrils from TEMPO-Mediated Oxidation of Date Palm Waste: Preparation, Characterization, and Reinforcing Potential
Source: Nanomaterials (Basel). 2022 Dec 26;13(1):126. doi: 10.3390/nano13010126 (PMC9824203; doi:10.3390/nano13010126)
Supplement: Supplementary file 1 [file nanomaterials-13-00126-s001.zip › nanomaterials-2109730-supplementary.pdf]

Supplementary Materials

Lignin-Containing Cellulose Nanofibrils from TEMPO-mediated Oxidation of Date Palm Waste: Preparation, Characterization, and Reinforcing Potential

Amira Najahi [1], Quim Tarrés [2], Pere Mutjé [2], Marc Delgado-Aguilar [2,*], Jean-Luc Putaux [3] and Sami Boufi [1,*]

[1] University of Sfax, LMSE, Faculty of Science, BP 802 – 3018 Sfax, Tunisia
[2] LEPAMAP-PRODIS Research Group, University of Girona. C/ Maria Aurèlia Capmany, 61 – 17003 Girona, Spain
[3] Université Grenoble Alpes, CNRS, CERMAV, F-38000 Grenoble, France
* Correspondence: m.delgado@udg.edu (M.D.-A.); sami.boufi@fss.rnu.tn (S.B.)

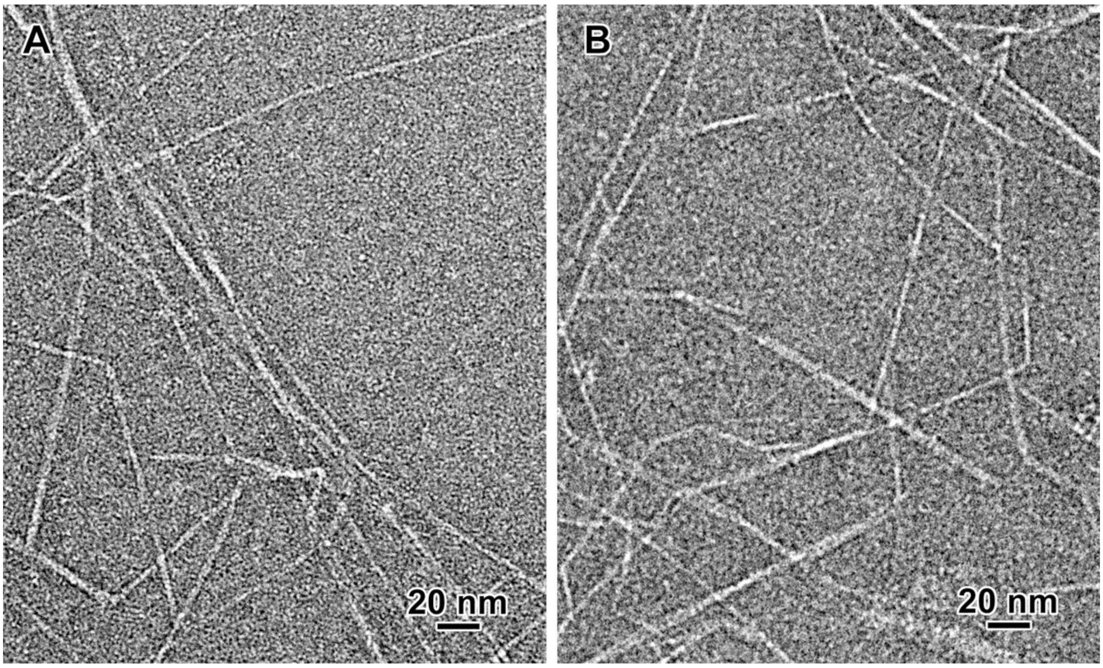

Figure S1. High-magnification TEM images of negatively stained preparations from LCNF-800 (A) and LCNF-1200 (B) showing bundled and individual cellulose nanofibrils.
